# Case Report: A novel TTN gene variant and a concurrent rare COL4A4 gene variant in a Chinese patient with dilated cardiomyopathy

**DOI:** 10.3389/fcvm.2025.1668842

**Published:** 2025-11-11

**Authors:** Shan Han, Ying-Yi Zhang, Jie Geng

**Affiliations:** Tianjin Key Laboratory of Cardiovascular Emergency and Critical Care, Department of Cardiology, Tianjin Municipal Science and Technology Bureau, Chest Hospital, Tianjin University, Tianjin, China

**Keywords:** TTN gene variant, dilated cardiomyopathy, rare variants, left ventricular reverse remodeling, case report

## Abstract

An estimated 30%–50% of dilated cardiomyopathy (DCM) cases are attributable to genetic factors, with titin (TTN) mutations constituting the most prevalent genetic etiology, accounting for 20%–25% of hereditary DCM cases. The majority of pathogenic TTN variants are heterozygous truncating mutations (TTNtv), including frameshift, nonsense, and canonical splice-site variants. Alport syndrome (AS) represents the second most common monogenic cause of end-stage kidney disease (ESKD). While COL4A5 gene variants cause X-linked AS, mutations in COL4A3 or COL4A4 genes (both located on chromosome 2) are associated with autosomal recessive AS, autosomal dominant AS, and thin basement membrane nephropathy. We present a unique case featuring both a novel TTN variant and a rare COL4A4 mutation in a DCM patient. This dual rare variant presentation is clinically exceptional and may contribute to expanding the genetic landscape of DCM and informing future investigations into genotype-phenotype correlations between TTN mutations and DCM pathogenesis.

## 1 Introduction

Dilated cardiomyopathy (DCM) represents a primary myocardial disease characterized by left ventricular (LV) dilation and impaired systolic function in the absence of significant coronary artery disease or abnormal loading conditions (1). The familial form is clinically defined by either: (1) ≥2 affected first- or second-degree relatives meeting diagnostic criteria, or (2) a first-degree relative with autopsy-confirmed DCM who experienced sudden cardiac death before age 50 (1). Genetic studies have identified >50 disease-associated genes encoding proteins involved in cytoskeletal integrity, nuclear envelope function, mitochondrial homeostasis, and calcium handling (2). Of these, TTN stands as the most frequent genetic determinant, accounting for 20%–25% of familial cases. TTN truncating variants (TTNtv) demonstrate particular clinical relevance, correlating with impaired LV reverse remodeling (LVRR) and heightened susceptibility to both atrial and ventricular arrhythmias (1).

Alport syndrome (AS) manifests as an inherited type IV collagen disorder featuring the classic triad of progressive glomerulopathy, sensorineural hearing loss, and ocular abnormalities. The X-linked form (COL4A5 mutations) predominates (80% of cases), while autosomal recessive inheritance (COL4A3/COL4A4) accounts for 15%. Rare autosomal dominant cases (5%) similarly arise from COL4A3/COL4A4 variants (3).

Emerging research continues to expand the spectrum of TTN variants in DCM populations. Our team previously reported a novel heterozygous variant NM_001267550.2: c.6790+3A>G in exon 29 of the TTN gene (4). We now describe a genetically complex DCM case featuring both a novel TTN variant and concurrent pathogenic mutation associated with Alport syndrome, representing an unusual instance of compound genetic cardiomyopathy.

## 2 Case presentation and clinical course

**Figure 1:**
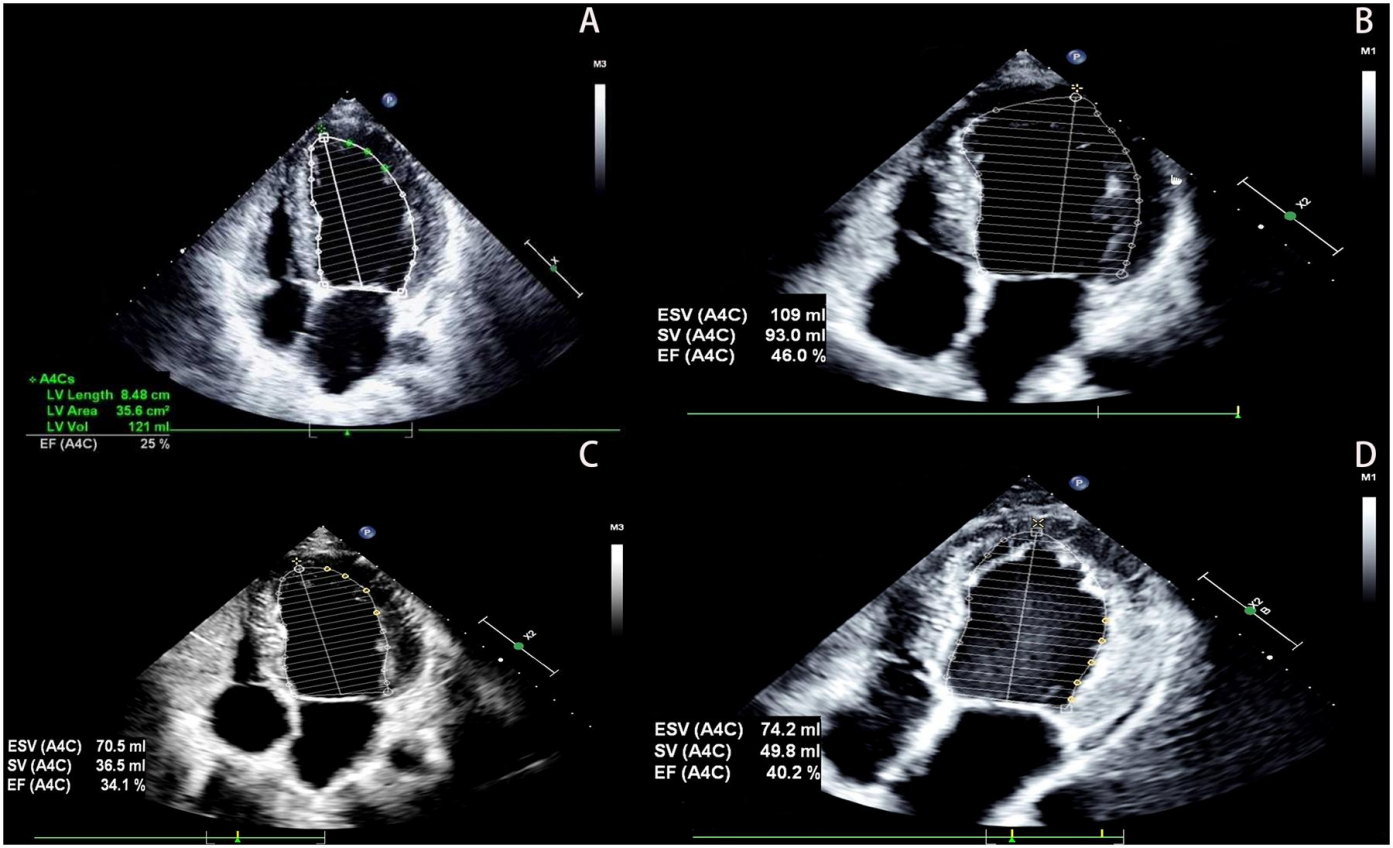
The patient's echocardiography results. **(A)** Show the echocardiographic result of the patient's heart at hospitalization (2022). **(B)** Show the echocardiographic result of the patient's heart at 1 year follow-up (2023). **(C)** Show the echocardiographic result of the patient's heart at 2 years after discharge, post-infection (2024). **(D)** Show the echocardiographic result of the patient's heart at 3 years follow-up (2025).

The proband was a 33-year-old man who presented to the outpatient department in May 2022 due to a 12-day history of shortness of breath. He had no history of hypertension, diabetes, hyperlipidemia, or other diseases, and denied smoking, drug use, or alcohol abuse. There was no family history of cardiac disease or sudden cardiac death at a young age. His blood pressure was 125/79 mmHg with a heart rate of 79 bpm. Electrocardiography (EKG) showed sinus rhythm, incomplete left bundle branch block, and left anterior fascicular block. Chest CT scan revealed left ventricular enlargement. Echocardiography demonstrated: left ventricular end-diastolic diameter (LVED) of 68 mm, left atrial diameter (LAD) of 46 mm, right atrial diameter (RAD) of 36 mm, right ventricular end-diastolic diameter (RVD) of 32 mm, interventricular septal thickness at diastole (IVSd) of 9 mm, left ventricular posterior wall dimensions (LVPWd) of 9 mm, and left ventricular ejection fraction (LVEF) of 25% (Figure 1A). The echocardiogram also showed myocardial thinning with non-compacted layer thickening. The patient was diagnosed with heart failure and treated with digoxin, furosemide, spironolactone, and sacubitril/valsartan. After one week without symptom improvement, he was hospitalized.

**Figure 2:**
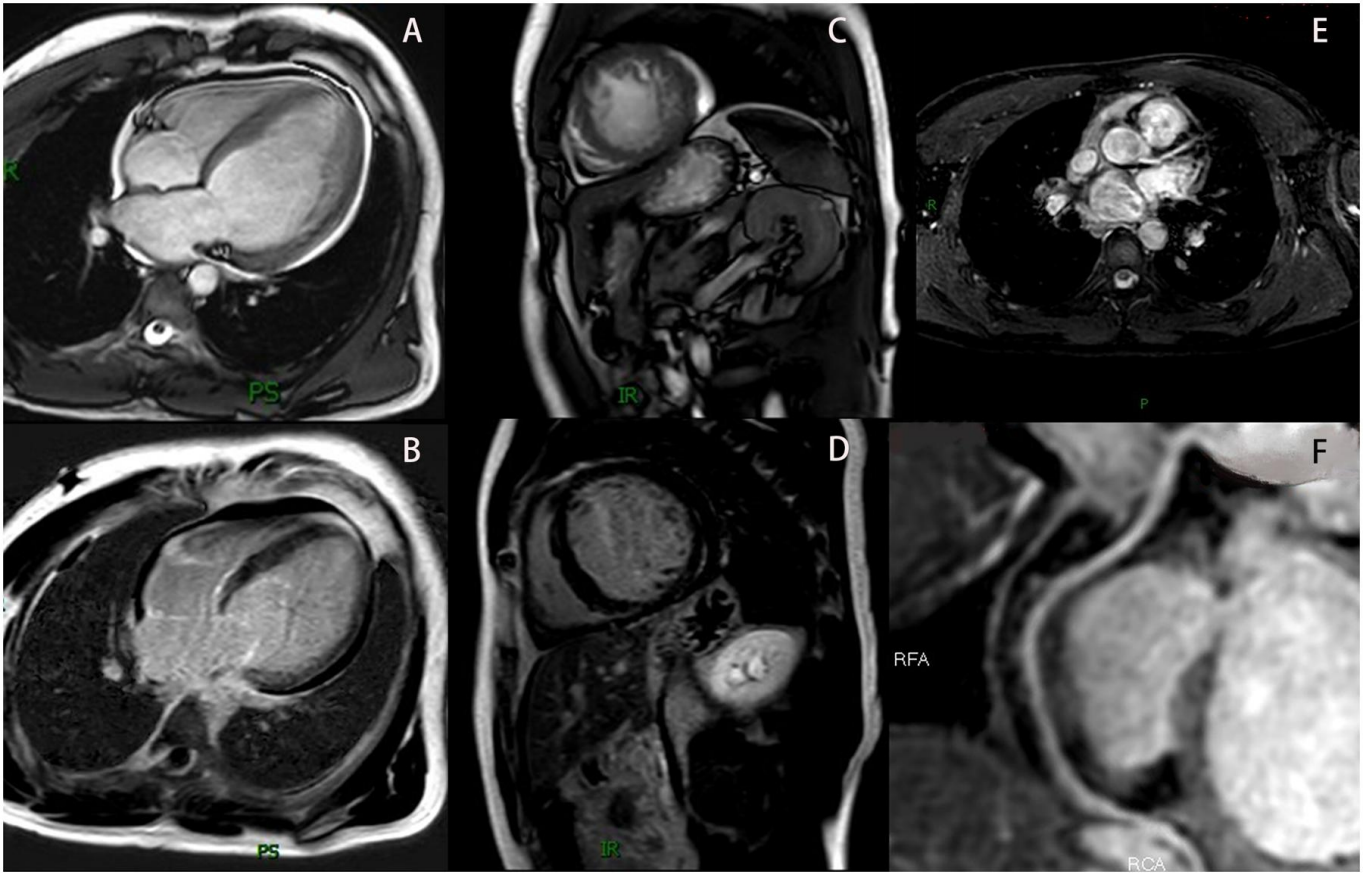
The patient's MRI results. **(A)** Show the cardiac MRI four-chamber image. **(B)** Show the LGE image in the four-chamber view. **(C)** Show the cardiac MRI short-axis image. **(D)** Show the LGE image in the short-axis view. **(E)** Show the LAD in the CMRA (The LCX was not visualized due to its diminutive caliber). **(F)** Show the RCA in the CMRA. MRI, magnetic resonance imaging; LGE, late gadolinium enhancement; LAD, left anterior descending artery; LCX, left circumflex artery; RCA, right coronary artery coronary; CMRA, coronary magnetic resonance angiography.

To identify the cause of heart failure, additional examinations were performed. Laboratory tests showed hyperlipidemia (cholesterol 5.30 mmol/L, LDL-C 3.69 mmol/L), elevated B-type natriuretic peptide (BNP 479.91 pg/mL), and positive urine occult blood (+). Other tests including complete blood count, coagulation profile, D-dimer, liver function, kidney function, blood glucose, electrolytes, thyroid function, iron studies, autoimmune markers, and urinary microalbumin-to-creatinine ratio (ACR) were normal. A 24 h Holter EKG showed occasional atrial premature contractions, ventricular premature beats, and short-duration ventricular tachycardia. Coronary magnetic resonance angiography (CMRA) revealed no obstructive coronary artery disease (Figures 2E,F). Cardiac magnetic resonance imaging (MRI) demonstrated: enlarged left ventricular end-diastolic volume (EDV) of 179.9 mL, LVED of 60 mm, depressed LVEF of 21%, reduced cardiac output (CO) of 2.8 L/min, and hypertrabeculation with wall thinning (Figures 2A,C). T1 mapping values and extracellular volume were normal, with no significant perfusion reduction during first-pass myocardial perfusion or abnormal enhancement on delayed imaging (Figures 2B,D).

Based on these findings, DCM was diagnosed. Although genetic testing was recommended for further clarification, the patient declined due to financial constraints. After 6 days of treatment with spironolactone, metoprolol, sacubitril/valsartan, and recombinant human brain natriuretic peptide, his condition improved. At discharge, he was prescribed spironolactone, metoprolol, and sacubitril/valsartan. Follow-up showed stable symptoms, and one year later, echocardiography revealed LVED of 60 mm, LAD of 47 mm, and LVEF of 46% (Figure 1B). Vericiguat was added to his medication regimen.

Two years after initial presentation (March 2024), the patient was readmitted for worsening dyspnea following infection. Echocardiography showed: LVED 57 mm, LAD 33 mm, RAD 31 mm, RVD 31 mm, and LVEF 34% (Figure 1C). He responded well to intravenous recombinant human brain natriuretic peptide and was discharged after 4 days. He maintained treatment with spironolactone, metoprolol, sacubitril/valsartan, and vericiguat, with regular follow-up showing stable NYHA class II symptoms, improved dyspnea, and increased exercise tolerance. At the 3-year follow-up, echocardiography demonstrated: LVED 58 mm, LAD 36 mm, RAD 35 mm, RVD 33 mm, and LVEF 40% (Figure 1D). Laboratory tests showed persistent microscopic hematuria (+) without proteinuria and normal renal function (Clinical parameters shows in Table 1).

**Table 1:** Clinical parameters of the proband.

Clinical parameters	First visit	Follow-up at 1 year	Follow-up at 2 years (post-infection)	Follow-up at 3 years
Echocardiographic parameters				
LAD (mm)	46	47	33	36
LVED (mm)	68	60	57	58
LVEF (%)	25	46	34	40
Blood test results				
WBC (*10^9 ^/L)	5.43	10.52	5.68	4.61
RBC (*10^12 ^/L)	5.70	4.91	4.26	4.63
HGB (g/L)	173	149	133	145
PLT (*10^9 ^/L)	223	193	234	233
BNP (pg/mL)	479.91	23.01	<10.00	38.63
BUN (mmol/L)	6.70	5.20	6.00	7.90
SCr (umol/L)	91.00	92.00	105.00	85.05
UA (umol/L)	331.00	259.00	227.00	172.00
K^+^	5.00	4.22	3.98	3.84
Na^+^	141.30	140.50	141.00	140.10
CL^+^	106.10	104.60	103.50	103.90
ALB (g/L)	41.50	41.20	42.20	37.00
TBIL (umol/L)	30.90	14.75	23.56	15.60
DBIL (umol/L)	9.60	1.53	1.44	7.40
ALT (u/L)	59.40	35.30	20.20	15.30
AST (u/L)	34.00	24.10	17.80	13.10
FBG (mmol/L)	4.44	5.09	4.42	5.10
TC (mmol/L)	5.30	5.07	3.17	3.31
TG (mmol/L)	1.31	1.09	0.55	0.62
LDL-C (mmol/L)	3.69	3.40	1.48	1.74
HDL-C (mmol/L)	1.39	1.38	1.51	1.44
CK-MB (u/L)	18.00	13.00	2.46	2.05
CK (u/L)	74.00	92.00	112.00	386.00
cTnT (ng/mL)	0.014	0.009	0.010	0.007
Urine occult blood	+	+	+	+

LAD, left atrial diameter; LVED, left ventricular end-diastolic diameter; LVEF, left ventricular ejection fraction; WBC, white blood cell count; RBC, red blood cell count; HGB, hemoglobin; PLT, platelet count; BNP, B-type natriuretic peptide; BUN, blood urea nitrogen; SCr, serum creatinine; UA, uric acid; K+, potassium; Na+, sodium; CL+, chloride; ALB, albumin; TBIL, total bilirubin; DBIL, direct bilirubin; ALT, alanine aminotransferase; AST, aspartate aminotransferase; FBG, Fasting Blood Glucose; TC, total cholesterol; TG, Triglycerides; LDL-C, low-density lipoprotein cholesterol; HDL-C, High-Density Lipoprotein Cholesterol; CK-MB, creatine kinase—myocardial band; CK, creatine kinase; cTnT, cardiac Troponin T.

**Figure 3:**
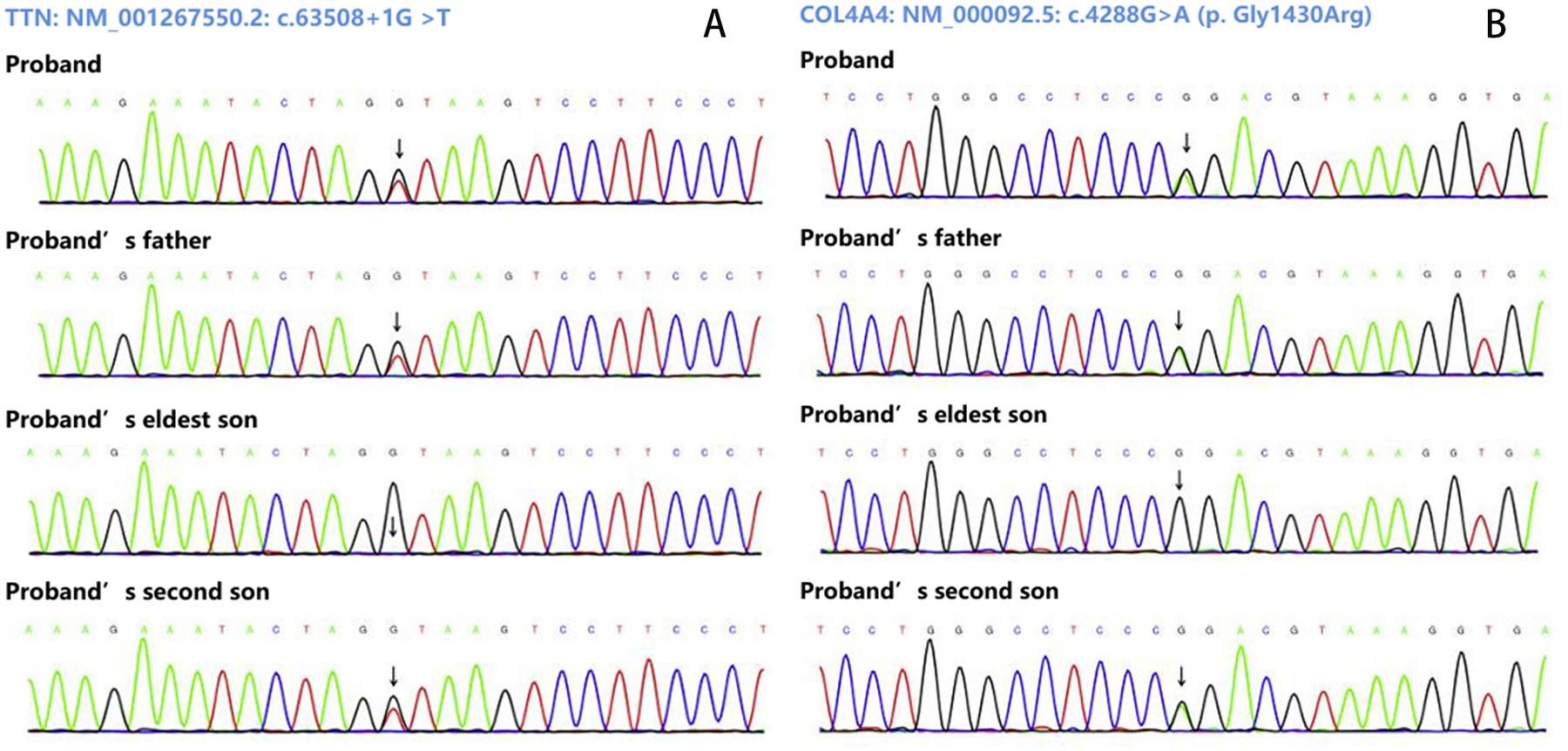
Sanger sequencing results. **(A)** Show the variant (NM_001267550.2: c.63508+1G>T of TTN gene. **(B)** Show the variant NM_000092.5: c.4288G>A [p. Gly1430Arg] of COL4A4gene.

**Figure 4:**
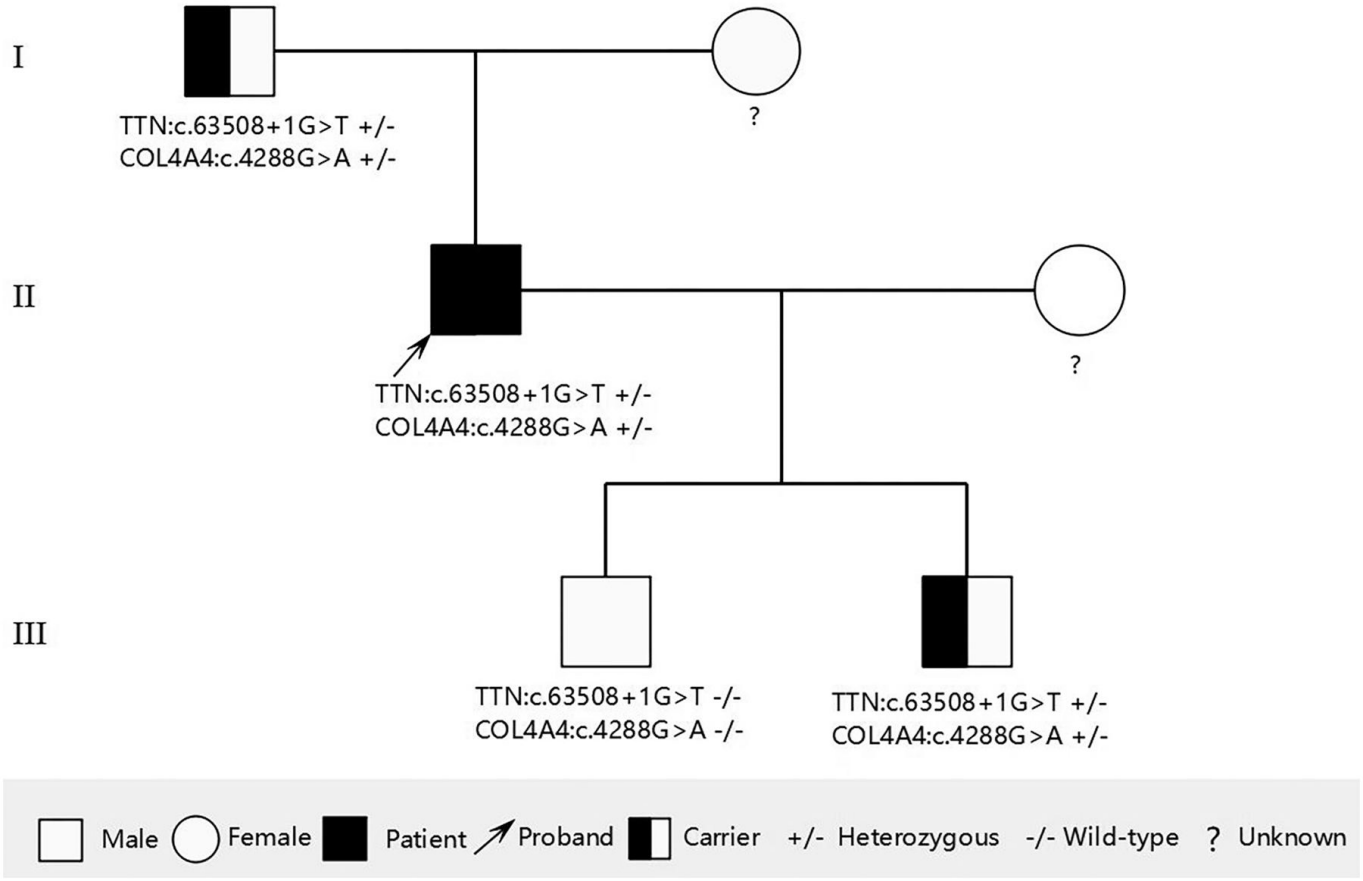
Pedigree of the family showing the genotypes of the proband and affected relatives. Male is represented by a square; female is represented by a circle.

During the third-year follow-up (March 2025), the proband consented to genetic testing. Following ethical approval and written informed consent, target-region capture and next-generation sequencing were performed on all exons and flanking regions. Rare and known variants were confirmed by Sanger sequencing, with cascade screening of available relatives. Genetic analysis identified: 1) a novel heterozygous splicing variant (NM_001267550.2: c.63508+1G>T) in TTN exon 305, and 2) a heterozygous missense variant [NM_000092.5: c.4288G>A (p. Gly1430Arg)] in COL4A4 exon 45. The father carried both variants but declined further evaluation. Of two sons, one carried both mutations while the other had neither. The mother declined genetic testing (Figures 3, 4).

## 3 Discussion

Titin, the largest human protein, serves as an essential structural and functional component of all striated muscles. This giant molecule acts as a bidirectional molecular spring that regulates muscle contraction and relaxation, while also participating in sarcomere organization, force transmission, mechanotransduction, and cellular signaling pathways. Structurally, each titin molecule spans half a sarcomere, with its N-terminus anchored in the Z-disc and C-terminus positioned at the M-line. In humans, titin is encoded by the TTN gene, which consists of 364 exons. Through extensive differential splicing—particularly in the I-band region—this single gene gives rise to multiple tissue-specific and developmentally-regulated isoforms. When considering DCM etiology, causative factors can be broadly categorized as genetic or non-genetic, with titin-truncating variants (TTNtv) representing the most common genetic predisposition (5).

TTNtv, including reading frameshifting insertions and deletions, premature termination codons, and splice site mutations, are estimated to account for 15%–25% of inherited DCM cases (6). The proband carries a heterozygous splice-site variant (c.63508+1G>T) in the TTN gene. This variant is absent in major population databases (1000 Genomes, ESP6500, ExAC) and was not detected in cardiomyopathy patients or controls within the Baisonovo local population database. Computational predictions strongly support a splicing impact: SpliceAI score: 0.99 (range 0–1; >0.2 indicates likely splicing disruption), and dbscSNV scores: ADA = 1.0, RF = 0.952 (scores >0.6 predict altered splicing, with higher values increasing confidence). The variant is not reported in ClinVar or HGMD and affects the canonical +1 splice site of exon 305. In silico analysis suggests this variant may induce a 76 bp deletion in exon 305, potentially leading to a truncated protein.

According to the American College of Medical Genetics and Genomics (ACMG) guidelines (7), canonical splice-site variants (e.g., ±1/2 positions) are presumed to disrupt gene function via nonsense-mediated decay (NMD) or nonfunctional transcripts. This variant is classified as “Likely Pathogenic (LP)” for DCM because it meets the following criteria: PVS1(null variant) and PM2 (absent from controls in Exome Sequencing Project, 1000 Genomes or ExAC). However, there is currently no familial segregation or functional evidence to further support its pathogenicity. Further evidence could support reclassification to Pathogenic (P).

Compared to DCM patients without other genetic diagnoses, those with TTNtv variants have worse LV dysfunction and LV dilation. However, among genetic causes of DCM, TTNtv patients are thought to have somewhat milder disease (6). TTN-related cardiomyopathies have a high potential for LVRR in contrast to other genetic cardiomyopathies, irrespective of other acquired etiologies (8). Truncating variants in the TTN gene mutation location in the predominantly adult splice TTN isoforms, with respect to A band or Cronos, does not impact on clinical phenotypes or outcomes. LVRR is frequent with medical therapy, but LV systolic function can deteriorate in the long term (9). Further, TTN mutations increase the risk of atrial and ventricular tachyarrhythmias (1). Malignant ventricular arrhythmia predominantly occurs in patients with severe LV systolic dysfunction and supports recommendations for prophylactic implantable cardioverter defibrillators in patients with advanced disease (9).

As observed in our previously reported cases (4), this proband similarly demonstrated LVRR following pharmacological therapy, with excellent short-term therapeutic efficacy. Although transient cardiac function deterioration occurred post-infection (March 2024), LVEF was rapidly restored with appropriate treatment. He remains under close surveillance to detect atrial and ventricular arrhythmia and LV systolic dysfunction recurrence following initial LVRR.

Left ventricular non-compaction (LVNC) is characterized by a prominent trabecular meshwork and extensive intertrabecular spaces that communicate directly with the ventricular cavity. It has been primarily classified as a congenital cardiomyopathy. The pathogenesis of LVNC has traditionally been attributed to an underdeveloped myocardial layer, believed to result from intrauterine arrest of the compaction process (10). Recent data demonstrate that LVNC is common among healthy individuals. Non-compaction is consistently identified in 1.5%–8% of athletes and in approximately 8% of healthy pregnant women. Among individuals with cardiomyopathy, LVNC may present as a coexisting phenotype, most frequently with DCM but also with hypertrophic cardiomyopathy (HCM). Recent guidelines have reclassified LVNC from a distinct cardiomyopathy to a phenotypic variant of DCM and/or HCM, recommending patient management according to disorder-specific guidelines (11).

Several studies have explored the genetic background of LVNC. It is now recognized that the genetic transmission of LVNC can also be autosomal recessive, X-linked, or mitochondrial, as reported in the literature (10). The strongest evidence supporting a genetic basis for LVNC comes from rare mutations in the mind bomb homolog 1 (MIB1) gene, a key component of the NOTCH signaling pathway that plays a critical role in normal cardiac embryologic development. Other putative LVNC variants often display mixed phenotypes both within and across pedigrees. This variant-specific phenotypic heterogeneity most commonly coincides with DCM but also occasionally with HCM, particularly apical HCM. In large cohorts, the spectrum of putative pathogenic mutations largely resembles that of DCM, with TTN variants being most common and multiple distinct gene ontologies identified, although HCM genes are also found (11). The proband presented with DCM combined with LVNC, suggesting that the newly identified TTN gene variant may be associated with the LVNC phenotype.

AS ranks as the second most prevalent monogenic cause of end-stage kidney disease (ESKD), following autosomal dominant (AD) polycystic kidney disease. AS is genetically characterized by pathogenic variants in genes encoding type IV collagen, an essential structural component of basement membranes in the kidneys, ears, and eyes. Variants in the COL4A5 gene cause X-linked AS. Variants of the COL4A3 or COL4A4 genes, both on chromosome 2, are responsible for autosomal recessive (AR) AS, AD AS, and thin basement membrane disease (TBMD) (12).

The proband carries a heterozygous missense variant c.4288G>A (COL4A4: p. Gly1430Arg) in the COL4A4 gene. Population frequency databases indicate this variant is rare (1000 Genomes: 8.3 × 10^−6^), ESP6500: not found, ExAC: 8.3 × 10^−6^). The variant was not detected in either hypertensive patients or controls within the Baisonovo local population database, though one carrier was identified with unavailable clinical data for phenotype correlation. Multiple bioinformatics tools consistently predict deleterious effects: SIFT (“D”), Polyphen-2 (“D”), MutationTaster_pred (“D”), VEST4 (0.939), REVEL (0.943), with an overall prediction profile of “6 deleterious/1 possibly damaging”. The substitution of nonpolar glycine with positively charged arginine at this evolutionarily conserved vertebrate residue suggests potential functional impact.

Database queries reveal conflicting interpretations in ClinVar [Pathogenic (1); Likely pathogenic (2); Uncertain significance (1)] while HGMD classifies it as “Disease-causing mutation”. Current evidence supports classification as LP, though additional clinical correlation and segregation data would strengthen this assessment.

Cohort studies have demonstrated that carriers may progressively develop proteinuria, chronic kidney disease (CKD), and ultimately ESKD. Female carriers with heterozygous COL4A5 variants and individuals with heterozygous COL4A3 or COL4A4 variants typically exhibit less severe renal involvement compared to AR AS patients or males with X-linked AS. Notably, hearing impairment and ocular abnormalities demonstrate lower prevalence and later onset in heterozygous carriers (12).

Current United States clinical guidelines for managing AS patients with CKD stages 1–4 (eGFR 15–90+ mL/min/1.73 m^2^) strongly recommend early initiation of either angiotensin-converting enzyme (ACE) inhibitors or angiotensin II receptor blockers (ARBs). While ACE inhibitors remain the preferred first-line therapy based on their marginally greater efficacy and more extensive evidence base, ARBs are commonly utilized as alternatives due to their superior tolerability profile. Furthermore, sodium-glucose cotransporter-2 (SGLT-2) inhibitors are increasingly being incorporated into treatment protocols. Although no AS-specific clinical trials exist, their inclusion is justified by well-documented renoprotective benefits in non-diabetic CKD populations, particularly in reducing progression to kidney failure (3).

The proband has developed microscopic hematuria and has been initiated on sacubitril/valsartan therapy. Close long-term monitoring of urinary analysis and renal function parameters is strongly recommended, specifically regular urine testing, renal function tests, blood pressure control (target <130/80 mmHg for CKD patients), and nephrology consultation is advised if proteinuria >300 mg/day or renal function decline occurs. This follow-up protocol is critical given the identified COL4A4 likely pathogenic variant and its association with progressive renal disease. Genetic counseling for at-risk family members should also be considered.

Given the established correlation between TTN truncating variants and progressive cardiac deterioration—compounded by the potential additive effects of multiple genetic variants—this patient faces an elevated risk of adverse clinical outcomes. Current management should anticipate possible future requirements for advanced heart failure therapies, including: primary prevention implantable cardioverter-defibrillator (ICD) for malignant arrhythmia prophylaxis, mechanical circulatory support as bridge-to-decision therapy, evaluation for orthotopic heart transplantation consideration. The patient remains under rigorous cardiological surveillance.

In recent years, advancements in phenomics have shifted research focus from the simplistic “single gene variant → single disease phenotype” model toward recognizing the complex interplay of “multiple gene variants + environmental factors → entire phenome network.” The international scientific community acknowledges that phenomic studies can elucidate the mechanisms underlying the relationships among phenotypes, genes, and environmental influences (13). The connection between genetic variations and phenomics serves as a cornerstone of modern life sciences, particularly in precision medicine. For this patient, clarifying the relationship between genetic variants and phenotypic manifestations—especially regarding the correlation between familial genetic variants and observed phenotypes—requires further investigation through phenomic studies.

## 4 Conclusion

This case report describes a rare instance of DCM associated with two pathogenic genetic variants, including a novel TTN mutation (c.63508+1G>T) not previously documented in the literature. Our findings contribute to the expanding genomic landscape of DCM pathogenesis, adding to both the Chinese and global databases of TTN-associated cardiomyopathy variants. Genetic counseling was provided to the family, with specific recommendations for prenatal testing in future pregnancies. Several limitations should be acknowledged: (1) the absence of functional studies to characterize the molecular consequences of these variants, (2) lack of mRNA-level validation of the splicing alteration, and (3) the inherent constraints of a single-case analysis. These findings underscore the importance of comprehensive genetic testing in DCM patients while highlighting the need for further research to establish the pathogenicity mechanisms of these rare variants.

## Data Availability

The data supporting this study are not publicly available due to patient privacy and ethical restrictions. However, upon reasonable request and with approval from the relevant ethics committee, the data can be obtained from the corresponding author.
